# Dysphagia caused by a lateral medullary infarction syndrome (Wallenberg's syndrome)

**Published:** 2012-07-31

**Authors:** Amine El Mekkaoui, Hanane Irhoudane, Adil Ibrahimi, Mounia El Yousfi

**Affiliations:** 1 Department of gastroenterology, Hassan II university hospital of Fez, Faculty of medicine and pharmacy of Fez - Sidi-Mohammed-Ben-Abdellah University of Fez, Morocco

**Keywords:** Dysphagia, lateral medullary infarction, Wallenberg's syndrome, deglutition

## Abstract

A 68-year-old man was referred to our hospital for a dysphagia evolving for 10 days. Clinical examination had found neurological signs as contralateral Horner's syndrome, ipsilateral palatal paresis, gait ataxia and hoarseness. Video-fluoroscopy showed a lack of passage of contrast medium to the distal esophagus. Esogastroduodenoscopy was normal. The cranial MRI had shown an acute ischemic stroke in the left lateral medullar region and the diagnosis of Wallenberg syndrome (WS) was established. WS remains an unknown cause of dysphagia in the clinical practice of the gastroenterologist.

## Introduction

Lateral medullary syndrome (LMS) or Wallenberg's syndrome (WS) is caused by a vascular event in the territory of the posterior inferior cerebellar artery or the vertebral artery [1]. In this report, we present a case of Wallenberg syndrome treated in our institute and we discuss its pathological and clinical features and review the related literature.

## Patient and observation

**Figure 1:**
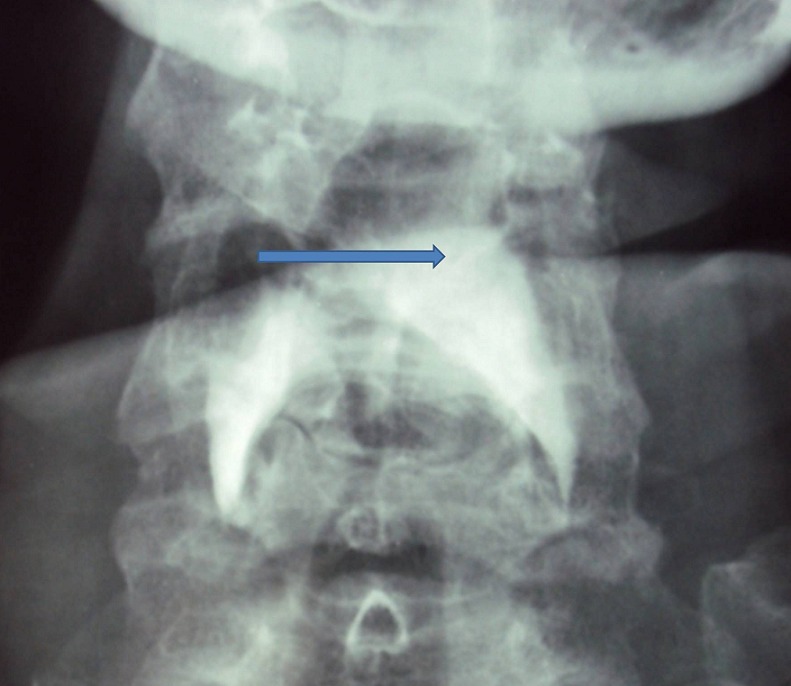
Video-fluoroscopy showing a lack of passage of contrast medium beyond the piriform sinuses towards the distal esophagus

**Figure 2:**
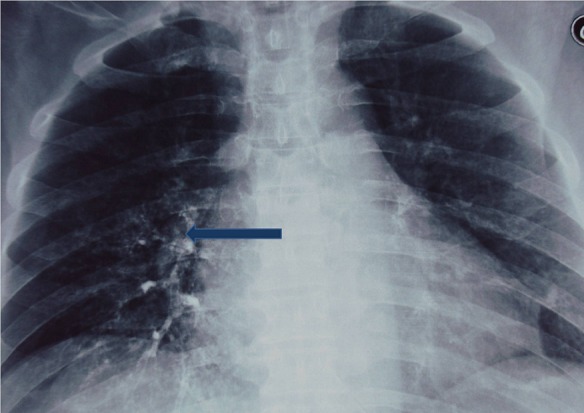
Chest X-ray showing a bronchial passage of the contrast agent

**Figure 3:**
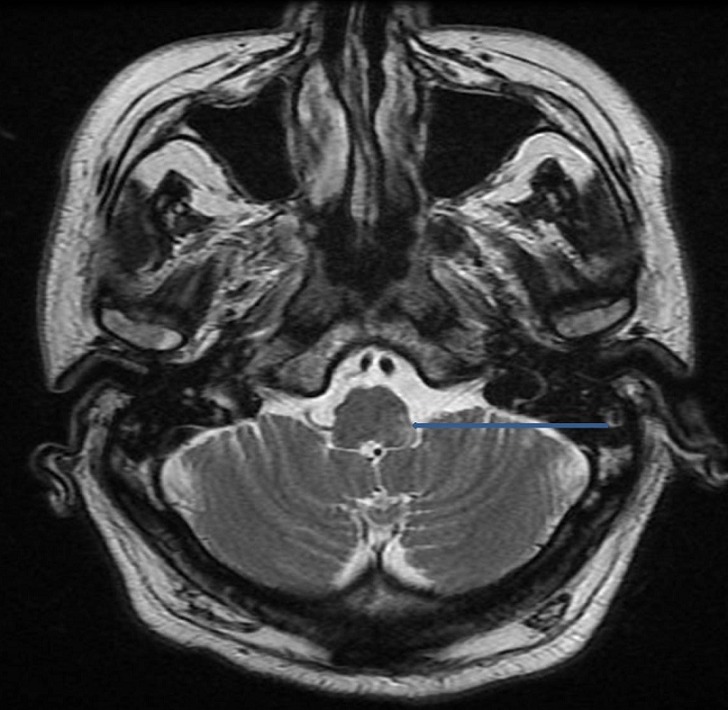
Cranial MRI showing a hyperintense lesion on T2 sequences in the right lateral medullary region

**Figure 4:**
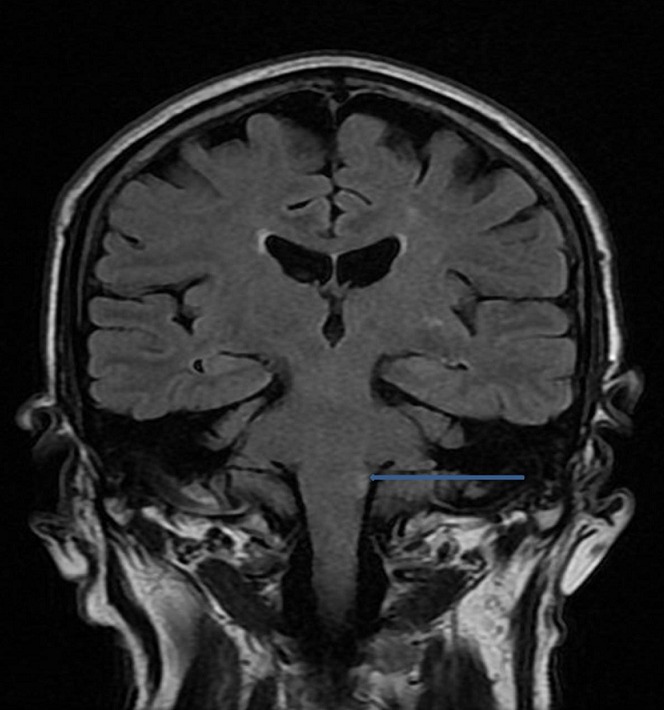
Coronal T2 FLAIR image showing hyperintense lesion in the right lateral medullary region

**Figure 5:**
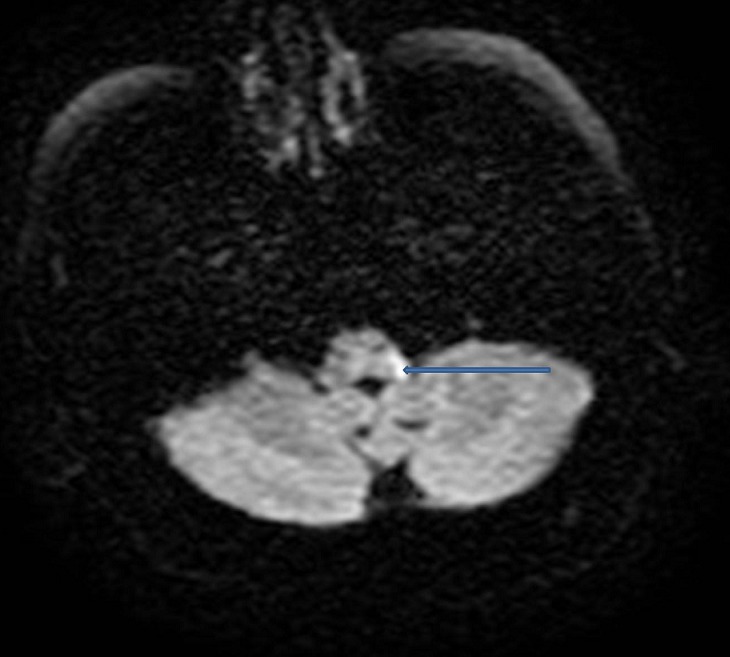
Diffusion-weighted axial MRI brain image showing the hyperintense

A 68-year-old man, with history of insulin-dependent diabetes was referred to our hospital for a severe dysphagia associated with false passages and nasal regurgitations evolving for 10 days. Clinical examination had found a blood pressure of 18/10 cmHg, with an irregular pulse. Cervical inspection and abdominal examination were normal. Neurological examination showed partial right Horner's syndrome, left palatal paresis and gait ataxia. Hoarseness was present. Video-fluoroscopy showed a lack of passage of contrast medium beyond the piriform sinuses towards the distal esophagus (Figure 1). Chest X-ray showed a bronchial passage of the contrast agent (Figure 2). An esogastroduodenoscopy revealed a normal oesophagus without mucous anomalies, the rest of the exploration was unremarkable. Because of neurological signs, a cranial MRI was obtained showing a hyperintense lesion on T2 and FLAIR sequences in the right lateral medullary region (Figure 3, Figure 4). This lesion was hyperintense on diffusion-weighted images (Figure 5) with a decline of the apparent diffusion coefficient (ADC) on the ADC map, compatible with an acute ischemic stroke. Cardiovascular exploration had found a complete arrhythmia by atrial fibrillation on hypertensive heart disease. The patient was managed with curative dose of anticoagulants and converting enzyme inhibitors. A percutaneous endoscopic gastrostomy (PEG) was established and oral feeding was progressed gradually until the dysphagia disappeared.

## Discussion

Dysphagia is common in the general population, and is generally due to mechanical obstruction, dysmotility or neurologic disease [2]. Swallowing is a complex motor event. Central control of swallowing is regulated by a central pattern generator (CPG) positioned dorsally in the solitary tract nucleus and neighboring medullary reticular formation. The CPG serially activates the cranial nerve motor neurons, including the nucleus ambigus and vagal dorsal motor nucleus, which then innervate the muscles of deglutition [1]. Swallowing difficulties may occur following cortical or brainstem infarction especially infarction of the swallowing centers in the rostral dorsolateral medulla which occurs in lateral medullary infarction (LMI) [3]. The clinical picture resulting from a LMI is known as Wallenberg's syndrome (WS) and results from the occlusion of the posterior inferior cerebellar artery or the vertebral artery [4]. Wallenberg's syndrome is typically presented with vertigo, dysarthria, nystagmus, ipsilateral ataxia, decreased facial sensation, Horner syndrome, decreased sensation on the contralateral body and diminished gag reflex [5]. However, the combination of signs and symptoms varies according to the site of the lesion. Severe dysphagia can complicate the clinical picture in 40% of patients with WS [6]. In this case the main symptom was dysphagia, accompanied by vertigo and gait ataxia. Dysphagia is not the main symptom at onset of Wallenberg's syndrome, so this case corresponds to an unusual presentation of this disease [4].

At the neurological level, bilateral medullary swallowing centers function as one integrated center, and the infarction of a portion of this center is sufficient to cause complete loss of swallowing [3]. So, in the present case, though the patient had a small unilateral brainstem lesion, dysphagia was severe. It is known that dysphagia is more prominent and lasts longer in WS patients than in hemispheric stroke patients [7]. Furthermore it is also a well-known fact that in the majority of patients with WS, this disorder is initially severe enough to require nonoral feeding, but often spontaneously recovers within 1 to 2 months after the stroke [8]. The second difference between WS and hemispheric stroke patients is related to the affected phase of the swallowing process. A higher incidence of symptoms related to the oral phase of swallowing is found in hemispheric stroke, whereas symptoms associated with the pharyngeal phase of swallowing and laryngopharyngeal paresis are mostly encountered in WS [8]. In fact, dysphagia in LMI, results from a contraction of the proximal pharyngeal and an absence of motor activity of the upper esophageal sphincter and proximal esophagus during the swallowing process [1].

Several mechanisms of LMI are described. It can be caused by a large artery disease (significant stenosis or occlusion of the relevant vertebral artery), cardiogenic embolism, small vessel disease (when patients have an infarction confined to a single perforator territory), arterial dissection, or undetermined etiology [9]. The continuous arrhythmia by atrial fibrillation explained the occurrence of the infarct at our patient.

Pre-mortem identification of LMI lesion became possible only after MRI was introduced. Nowadays, cranial MRI (T1, T2, and gadolinium-enhanced T1-weighted scans) is the gold standard for diagnosis of such lesions [6]. Otherwise, oropharyngeal dysphagia generally results from Neurological disorders or cancerous lesions. Given the advanced age and discretion, at the onset, of neurological signs in the present case, endoscopy was performed to remove an organic lesion of the upper esophagus and to eliminate contraindication of a possible PEG.

In LMI, dysphagia is managed using compensatory strategies: early nasogastric feeding, thickened fluids or percutaneous endoscopic gastrostomy feeding, followed by a progressive deglutition rehabilitation program. Some authors suggest the effectiveness of therapeutic repetitive transcranial magnetic stimulation [10]. In our case, we set up a PEG feeding at the beginning and then a progressive rehabilitation to food intake was established. Two months later, there was a net regression of dysphagia and the patient was able to eat normally.

## Conclusion

We report an atypical case of Wallenberg's syndrome, in which dysphagia was the main symptom at the onset. The research of neurological signs was helpful to suspect neurogenic origin especially when esogastroduodenoscopy was normal. The neurogenic origin of acute oropharyngeal dysphagia should be suspected in the practice of gastroenterology.

## References

[1] Martino R, Terrault N, Ezerzer F, Mikulis D, Diamant NE (2001). Dysphagia in a Patient With Lateral Medullary Syndrome: Insight Into the Central Control of Swallowing. Gastroenterology..

[2] Kuo P, Holloway RH, Nguyen NQ (2012). Current and future techniques in the evaluation of dysphagia. J Gastroenterol Hepatol..

[3] Vigderman AM, Chavin JM, Kososky C, Tahmoush AJ (1998). Aphagia due to pharyngeal constrictor paresis from acute lateral medullary infarction. Journal of the neurological sciences..

[4] Loaeza-del Castillo A, Barahona-Garrido J, Criales S, Chang-Menéndez S, Torre A (2007). Wallenberg's Syndrome: An Unusual Case of Dysphagia. Case Reports in Gastroenterology..

[5] Chen C-N, Khor G-T, Chen C-H, Huang P (2011). Wallenberg's Syndrome With Proximal Quadriparesis. The Neurologist..

[6] Kim JS (2003). Pure lateral medullary infarction: clinical-radiological correlation of 130 acute, consecutive patients. Brain..

[7] Ertekin C, Aydogdu I, Tarlaci S, Turman AB, Kiylioglu N (2000). Mechanisms of Dysphagia in Suprabulbar Palsy With Lacunar Infarct. Stroke..

[8] Aydogdu I, Ertekin C, Tarlaci S, Turman B, Kiylioglu N, Secil Y (2001). Dysphagia in Lateral Medullary Infarction (Wallenberg's Syndrome): An Acute Disconnection Syndrome in Premotor Neurons Related to Swallowing Activity?. Stroke..

[9] Lee MJ, Park YG, Kim SJ, Lee JJ, Bang OY, Kim JS (2012). Characteristics of stroke mechanisms in patients with medullary infarction. Eur J Neurol.

[10] Khedr EM, Abo-Elfetoh N (2010). Therapeutic role of rTMS on recovery of dysphagia in patients with lateral medullary syndrome and brainstem infarction. J Neurol Neurosurg Psychiatry..

